# Supplementary material for: Immune modulation by complement receptor 3-dependent human monocyte TGF-β1-transporting vesicles
Source: Nat Commun. 2020 May 11;11:2331. doi: 10.1038/s41467-020-16241-5 (PMC7214408; doi:10.1038/s41467-020-16241-5)
Supplement: Supplementary file 1 — Supplementary Information [file 41467_2020_16241_MOESM1_ESM.pdf]

Supplementary Information

Immune modulation by complement receptor 3 dependent human monocyte TGF-β1-transporting vesicles

Halder *et al.* 2020

Supplementary Figure 1: **Characterization of EVs isolated by different methods**

Supplementary Figure 2: **TGF-β1-transporting vesicle characterization**

Supplementary Figure 3: **iC3b binds to CR3**

Supplementary Figure 4: **KEGG enrichment analysis of MEV_{Ca} and $MEV_{Ca\text{-}s\beta G}$**

Supplementary Figure 5: **TGF-β1 is located on vesicles upon whole blood infection**

Supplementary Figure 6: **HUVEC controls**

Supplementary Figure 7: **FACS gating and blots**

Supplementary Table 1: **Primers**

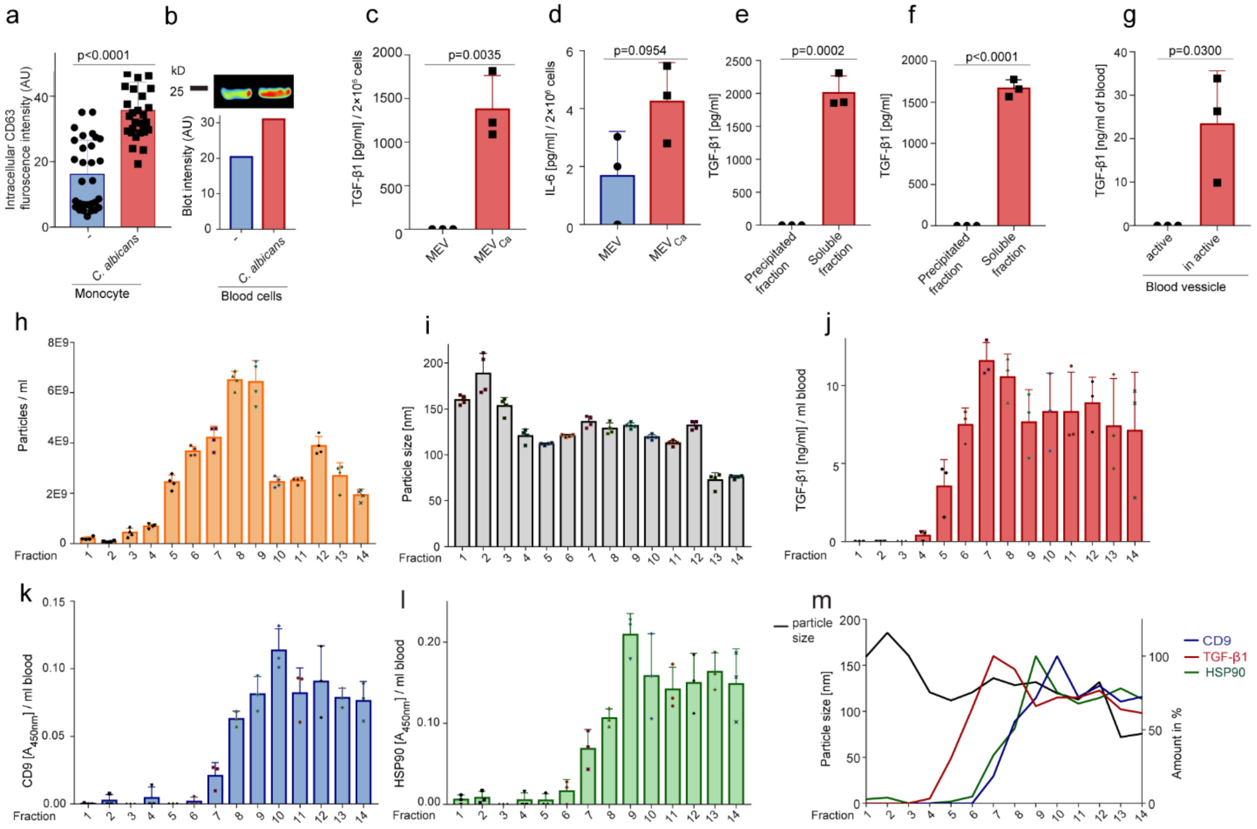

Supplementary Figure 1: Characterization of EVs isolated by different methods

(a) Intracellular vesicle marker protein—CD63 increases in *C. albicans*-infected monocytes as determined by confocal laser scanning microscopy (CLSM). Stained intracellular CD63 was measured by ZEN 2011 (data are presented as mean values +/- SD, p<0.0001, unpaired two tailed t-test, n=32 individual cells from 3 donors). **(b)** Intracellular CD9 protein increases in *C. albicans*-infected (1h) whole blood cells compared to untreated cells. Cells were lysed and CD9 detected by Western blot. n=3 experiments and donors. **(c)** TGF-β1 but not **(d)** IL-6 significantly increases in $MEVs_{Ca}$ compared to MEVs. Cytokines from were determined by sandwich ELISA (data in **c**, **d** are presented as mean values +/- SD, p=0.0035, p=0.0954, unpaired two tailed t-test, n=3 donors). EVs were isolated from 2×10^{6} uninfected or opsonized *C. albicans*-infected monocytes (MEVs or $MEVs_{Ca}$, respectively) by ultracentrifugation. Soluble recombinant TGF-β1 **(e)** as well as recombinant latent TGF-β1 **(f)** stay in solution after ExoQuick-TC precipitation (data in **e, f** are presented as mean values +/- SD, p=0.0002, p<0.0001, unpaired two tailed t-test, n=3 independent experiments). **(g)** TGF-β1 on blood vesicles of *C. albicans* infected mice (24 h) is predominantly inactive (latent form) (data are presented as mean values +/- SD, p=0.0300, unpaired two

tailed t-test, n=3 donors). Particle amount **(h)** and size **(i)** profiles of different fractions after size exclusion chromatography detected by DLSM (data are presented as mean values +/- SD, n=4 donors). EVs were isolated from 1 ml of human blood infected with 1×10^8 *C. albicans*. **(j)** TGF-β1 concentrations in fractions after size exclusion chromatography detected by ELISA. Distribution of CD9 **(k)** and HSP90 **(l)** on TGF-β1 particles in fractions obtained from size exclusion chromatography. CD9 and HSP90 proteins were detected on selected TGF-β1 particles by sandwich ELISA (in **j**, **k**, **l** data are presented as mean values +/- SD, n=3 donors). **(m)** Fractions 7 to 14 harbor TGF-β1 particles which carry CD9 and HSP90 vesicle markers and which range from 70-130 nm (data are presented as mean values, n=3 donors for CD9, TGF-β1, HSP90, n=4 for particle size).

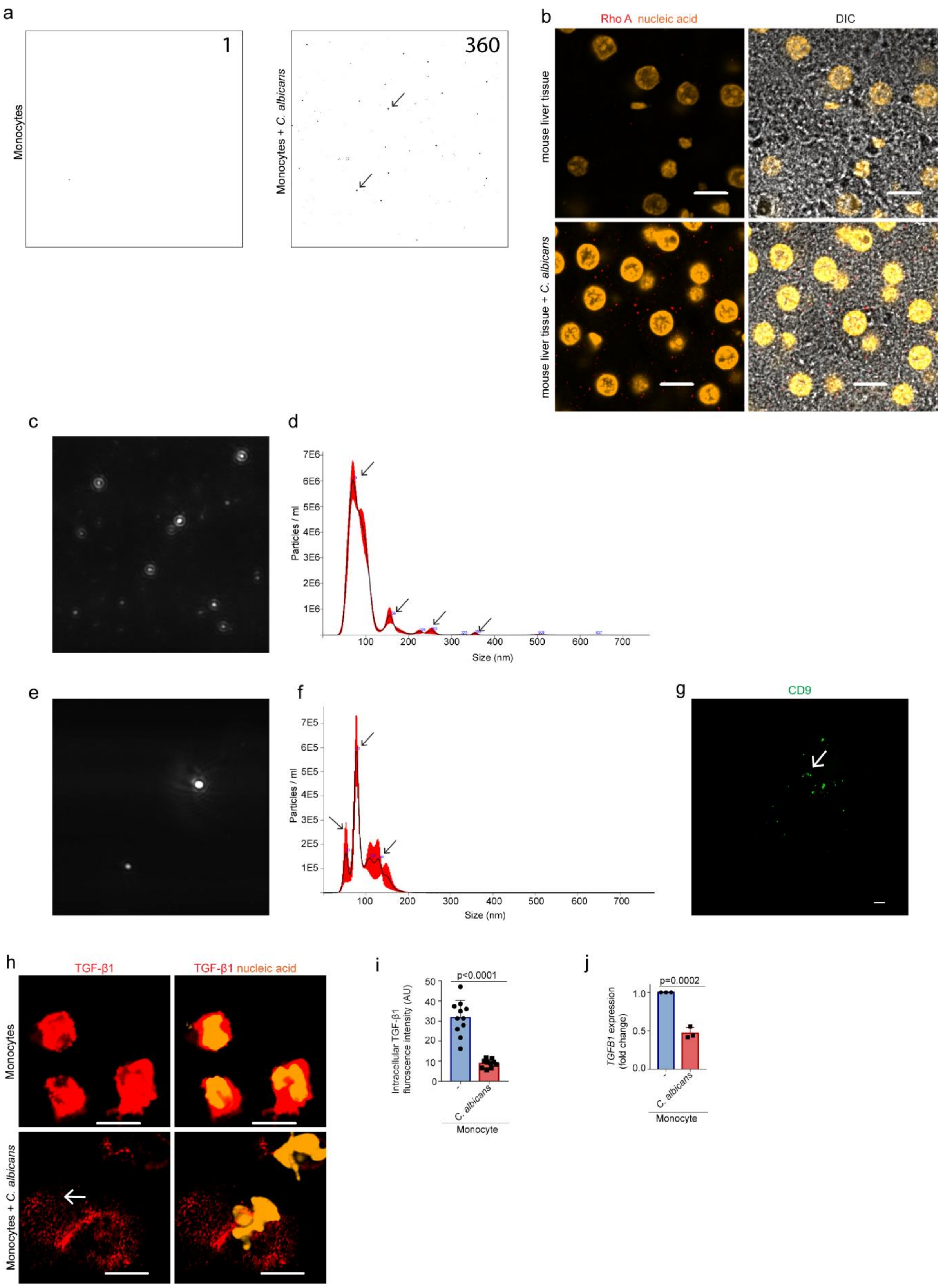

Supplementary Figure 2: TGF-β1-transporting vesicle characterization

(a) TGF-β1 is detected on $MEVs_{Ca}$ compared to MEVs. TGF-β1-transporting EVs were counted by using Image J software (n=4 donors). **(b)** Rho A-transporting vesicles appear frequently in liver tissue of *C. albicans* infected mice (24 h) as demonstrated by immunohistochemistry. Staining: Rho A and nucleic acids. Bars: 10 µm (Representative of n=3 donors). **(c)** TGF-β1-transporting vesicles tracked in DLSM show a **(d)** size distribution of predominantly 50-100 nm. **(**$BCEVs_{Ca}$) were isolated from 1×10^9 blood cells. TGF-β1-transporting vesicles were isolated from *C. albicans*-infected blood cell vesicles $BCEVs_{Ca}$ by using anti-TGF-β1 antibody coated beads and analyzed using NanoSight NTA 3.2 software. **(e)** CD9 & TGF-β1-transporting vesicles show a **(f)** size distribution predominantly below 100 nm as tracked in DLSM. **(g)** TGF-β1-transporting vesicles stain for CD9 using CLSM. Bars: 10 µm. CD9-transporting EVs were isolated from isolated TGF-β1-transporting vesicles using anti-CD9 antibody coated beads. Data in **c**, **d**, **e**, **f**, **g** are representatives of each n=3 independent experiments and donors. **(h)** Intracellular TGF-β1 is released from human infected monocytes on vesicles. Staining: TGF-β1 and nucleic acids. Bars: 10 µm. **(i)** Intracellular TGF-β1 measured with ZEN 2011 software (data are presented as mean values +/- SD, p<0.0001, unpaired two tailed t-test, n=11 individual cells from 3 donors). **(j)** TGF-β1 transcription is not upregulated in *C. albicans* infected monocytes as shown by comparative qPCR (data are presented as mean values +/- SD, p=0.0002, unpaired two tailed t-test, n= 3 donors).

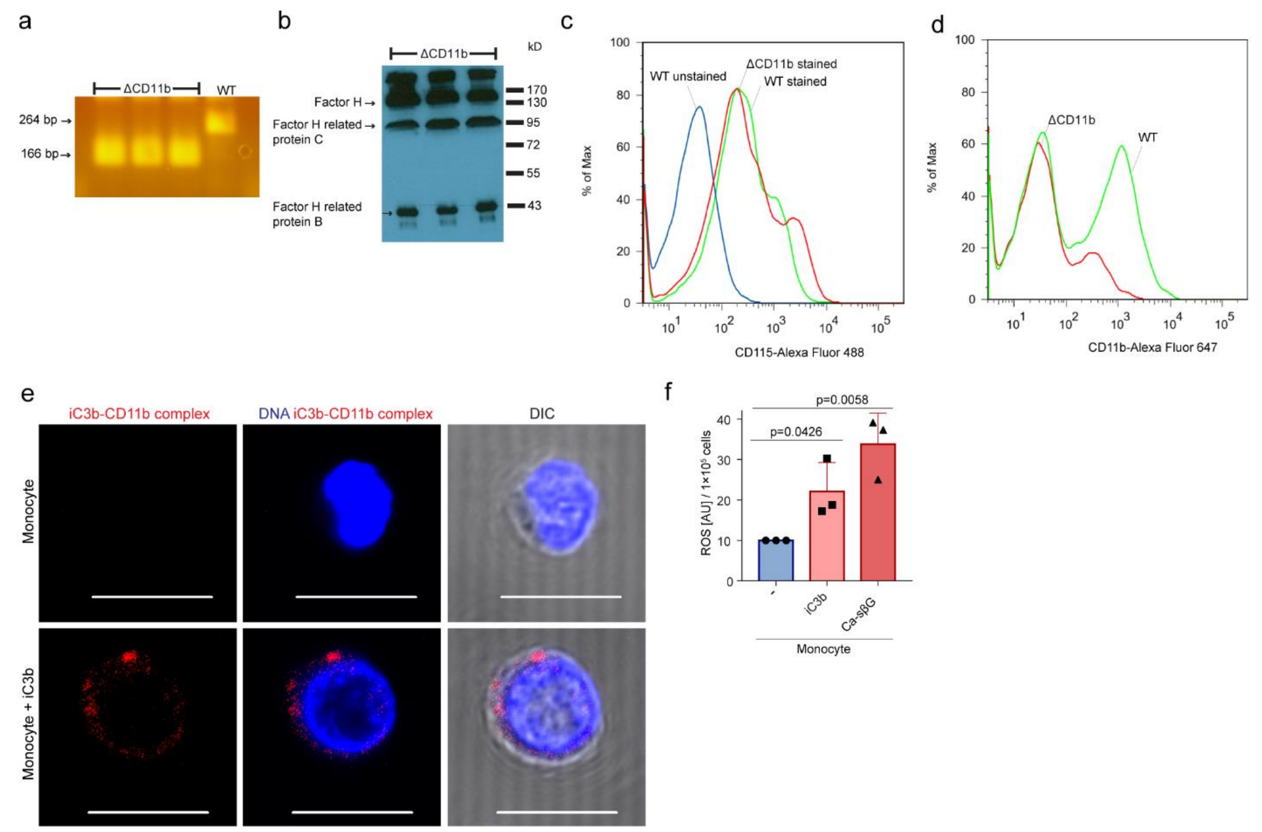

Supplementary Figure 3: iC3b binds to CR3

(a) Three CD11b KO mice show presence of the *ITGAM* gene determined by PCR followed by gel electrophoresis (n=3 animals) (see uncropped electrophoresis in Supplementary Fig. 7f). **(b)** Equal presence of complement Factor H in sera derived from three CD11b KO mice using western blot analysis. **(c)** Monocytes isolated from wild type and CD11b KO mice carry the monocyte marker CD115 in flow cytometry. **(d)** Monocytes from CD11b KO mouse lack the CD11b signals in contrast to monocytes from wild type mouse using flow cytometry. Monocytes were generated from mouse bone marrow derived stem cells.(see gating strategy in Supplementary Fig. 7b). **(e)** Soluble iC3b binds to CR3 on monocytes as detected by PLA. PLA staining: iC3b-CD11b complex by CLSM. Bars: 10 µm. The experiments **a**, **b**, **c**, **d**, **e** were confirmed with 3 independent donors. **(f)** Both iC3b and sβG induce ROS formation in human monocytes within 30 min. ROS formation was determined by CellROX staining in a fluorescence plate reader (data are presented as mean values +/- SD, p=0.0426, p=0.0058, unpaired two tailed t-test, n=3 donors).

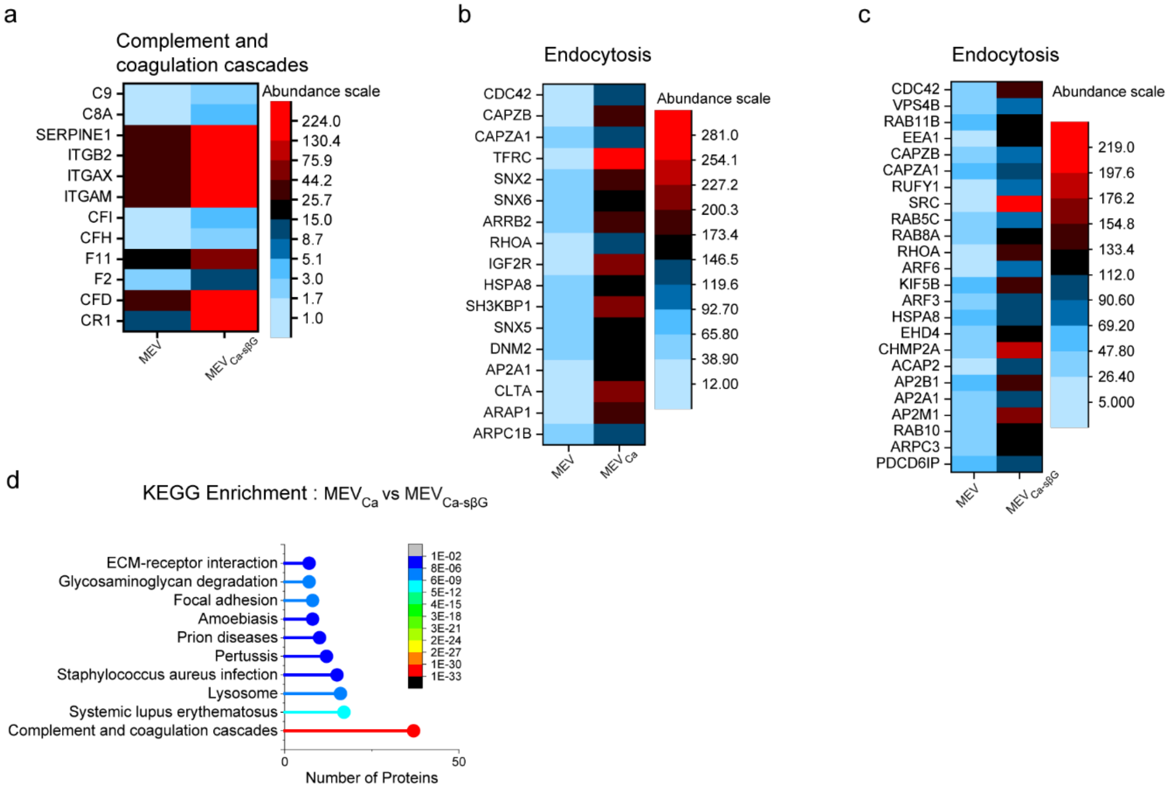

Supplementary Figure 4: KEGG enrichment analysis of MEV$_{Ca}$ and MEV$_{Ca\text{-}s\beta G}$

(a) Complement and coagulation proteins are more abundant in MEV$_{Ca\text{-}s\beta G}$ compared to MEVs. Heat map was obtained by Kyoto Encyclopedia of Genes and Genomes (KEGG) enrichment analysis. Analysis was performed of the higher protein content of MEV$_{Ca\text{-}s\beta G}$ of n=3 donors. **(b)** Endocytosis proteins are more present in MEV$_{Ca}$ and **(c)** MEV$_{Ca\text{-}s\beta G}$ than in MEVs. Heat maps were obtained by KEGG analysis. Analysis was performed for the higher protein content of MEV$_{Ca}$ and MEV$_{Ca\text{-}s\beta G}$ from n=3 donors. **(d)** KEGG analysis of MEV$_{Ca}$ vs MEV$_{Ca\text{-}s\beta G}$ upregulated proteins. Analysis was performed of the higher protein content of MEV$_{Ca\text{-}s\beta G}$ of n=3 donors.

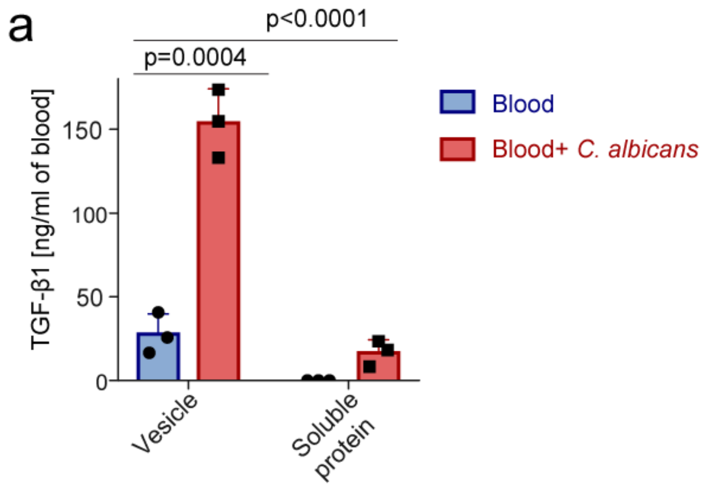

Supplementary Figure 5: TGF-β1 is located on vesicles upon whole blood infection

(a) TGF-β1-transporting vesicle release significantly increases in *ex vivo* whole blood infected with *C. albicans* (data are presented as mean values +/- SD, p=0.0004, unpaired two tailed t-test, p<0.0001, ordinary one-way ANOVA, n=3 donors). Significantly low concentrations of soluble TGF-β1 are detected by sandwich ELISA.

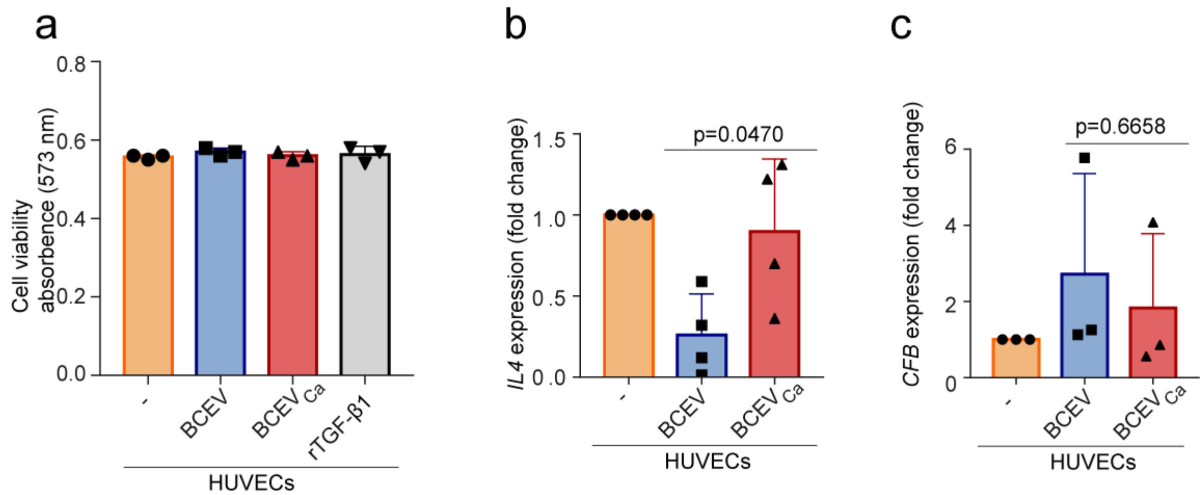

Supplementary Figure 6: HUVEC controls

(a) HUVEC's viability remains unchanged in response to incubation with BCEVs, BCEVs$_{Ca}$, or recombinant TGF-β1 as measured by cell titer blue assay (data are presented as mean values +/- SD, n=3 donors). **(b)** IL-4 expression in HUVECs is upregulated when the cells were treated with BCEVs$_{Ca}$ but not with BCEVs. HUVECs (3×10^7) were treated for 6 h with BCEVs or BCEVs$_{Ca}$ isolated from 5×10^8 blood cells. RNA was isolated and evaluated by comparative qPCR (data are presented as mean values +/- SD, p=0.047, unpaired two tailed t-test, n=3 donors). **(c)** No significant change is observed in complement factor B expression in HUVECs treated with BCEVs$_{Ca}$ or BCEVs (data are presented as mean values +/- SD, p=0.6658, unpaired two tailed t-test, n=3 donors).

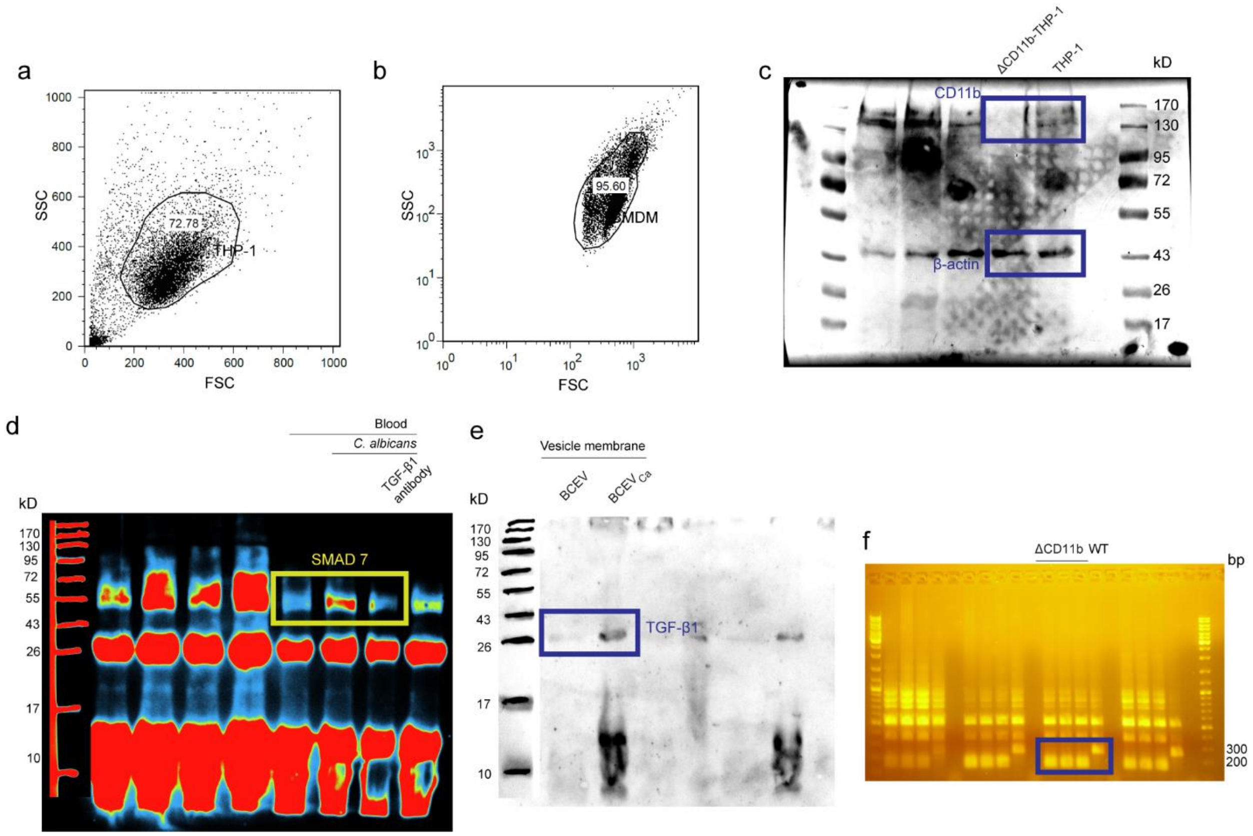

Supplementary Figure 7: FACS gating and blots

(a) Gating strategy for flow cytometry of THP-1 cell lines (for Fig. 4b). **(b)** Gating strategy for flow cytometry of bone marrow derived monocytes (for Supplementary Fig 3c and 3d). **(c)** Uncropped Western blot for the detection of CD11b and β-actin (for Fig. 4b). **(d)** Uncropped Western blot for the detection of SMAD7 (for Fig. 6e). **(e)** Uncropped Western blot for the detection of TGF-β1 (for Fig. 7e). **(f)** Uncropped gel electrophoresis for the detection of *ITGAM* gene (for Supplementary Fig 3a).

Supplementary Table 1: Primers

Primer for	forward	reverse
mouse *ITGAM* of C57BL/6	TGTTTTTACCCCTCCCTCCT	CCTTTGATCTCTCCCCACCT
mouse *ITGAM* of B6.129S4-Itgam$^{tm1Myd/J}$	TGTTTTTACCCCTCCCTCCT	TGATTCCCACTTTGTGGTTC
guide oligos	AAACCCGGGGGCCGAACCCCAGTAC	CACCGTACTGGGGTTCGGCCCCCGG
human *ACTB*	CTGTCTGGATTGGTGGTTCTATC	GATGGACCAGATTCGTCGTATTC
human *IL-1β*	CTCTCACCTCTCCTACTCACTT	TCAGAATGTGGGAGCGAATG
human IL-6	GGAGACTTGCCTGGTGAAA	CTGGCTTGTTCCTCACTACTC
human *TFG-β1*	GGCCTTTCCTGCTTCTCAT	CGTGGAGCTGAAGCAATAGT
human *LGALS1*	GAACATCCTCCTGGACTCAATC	GGTTCAGCACGAAGCTCTTA
human *IL-4*	GTTCTACAGCCACCATGAGAA	CCGTTTCAGGAATCGGATCA
human *Factor B*	GGGACCTGGAGATAGAAGTAGT	AGGGCAACGTCATAGTCATAAA
mouse *ACTB*	GAGGTATCCTGACCCTGAAGTA	CACACGCAGCTCATTGTAGA
mouse *IL-1β*	GGTGTGTGACGTTCCCATTA	ATTGAGGTGGAGAGCTTTCAG
