# Supplementary material for: Immune modulation by complement receptor 3-dependent human monocyte TGF-β1-transporting vesicles
Source: Nat Commun. 2020 May 11;11:2331. doi: 10.1038/s41467-020-16241-5 (PMC7214408; doi:10.1038/s41467-020-16241-5)
Supplement: Supplementary file 2 — Description of Additional Supplementary Files [file 41467_2020_16241_MOESM2_ESM.docx]

**Description of Additional Supplementary Files**

**File name:** Supplementary Video 1

**Description:** Real time formation and release of MEVsCa from monocytes in presence of opsonized C. albicans cells
